# Supplementary material for: PLOS Neglected Tropical Diseases 2014 Reviewer Thank You
Source: PLoS Negl Trop Dis. 2015 Feb 27;9(2):e0003621. doi: 10.1371/journal.pntd.0003621 (PMC4344223; doi:10.1371/journal.pntd.0003621)
Supplement: S1 Reviewer List — (PDF) [file pntd.0003621.s001.pdf]

PLOS Neglected Tropical Diseases would like to thank all those who reviewed on behalf of the journal in 2014:

Thomas A Ficht
Matthew Aardema
John Aaskov
Fernando Abad-Franch
Emmanuel Abatih
Syed Abbas
Ali AbdElrazek
Behnoush Abedi-Ardekani
Sören Abel
Fred Aboagye-Antwi
David Abraham
Patrícia Antonia Estima Abreu
Nadia Abuelezam
Amit Achhra
Thomas Achia
Hans Ackerman
Mercy Ackumey
Alvaro Acosta-Serrano
Gerardo Acosta
Ben Adams
Emily Adams
Linda Adams
David Addiss
Oluwatoyosi Adekeye
Monsuru Adeleke
Zach Adelman
Peter Adler
Emily Adrion
Farhat Afrin
Suneth Agampodi
Madhur Aggithaya
Javed Agrewala
Peter Agre
Patricia Aguilar
Tolulope Agunbiade
Fernán Agüero
Abdulhamid Ahmed
Be-Nazir Ahmed
Kamruddin Ahmed
Umesh Ahuja
Moses Aikins
Dora Akinboye
Robert Akins
Oladele Akogun
Jean Paul Akue
Hannah Akuffo
Hesham Al-Mekhlafi
Jorge Alarcon
John Alawneh
Rosana Alberici
Marco Albonico
Jared Aldstadt
James Alexander
Neal Alexander
Julio Aliberti
Matthew Aliota
Asad Ali
Innocent Ali
Mohammad Ali
Nahid Ali
Mohan Alladi
E. Kaitlynn Allen
Margaret Allen
Pascale Allotey
Rodrigo Almeida-Paes
Igor Almeida
Cristina Alonso-Vega
Wladimir Alonso
Luke Alphey
Benjamin Althouse
Jacqueline Alvarez-Leite
Lysangela Alves
Ananda Amarasinghe
Adeladza Amegah
Kei Amemiya
Mansoor Amiji
Rogiero Amino
Nana Ama Amissah

Yaw Amoako
Emmanuel Ampofo
Ahmad Amro
Burt Anderson
Kathryn Anderson
Robert Anderson
Tim Anderson
Katherine Anders
Bruno Andrade
Luciana Andrade
Katherine Andrews
Jon Andrus
Nestor Anez
Jussara Angelo
Andrea Angheben
Serge Ankri
Dhekra Annuzaili
Nicholas Anstey
Tiago Antao
Peter Anthamatten
Maria Aoki
Juan Aparicio
Ruslan Aphasizhev
Charles Apperson
Werner Apt
Byron Arana
Tania Araújo-Jorge
Catarina Araújo
Bruno Arca
Jorge Arevalo
Paul Arguin
Armando Arias
Larry Arlian
Peter Armbruster
Alejandro Armenti
Philip Armstrong
Michael Arndt
Benjamin Arnold
Catherine Arnold
Raffi Aroian
Luciana Arruda
Sridhar Arumugam
Kingsley Asiedu
Brenda Asiimwe-Kateera
Gemma Atkinson
Geoffrey Attardo
Herbert Auer
Steven Ault
Vicky Avery
Guillermina Avila
Tatjana Avšič-Županc
Ali Ayadi
Abdu Azad
Aishah Azil
Germán Añez
Marina BENTIVOGLIO
Christophe BOETE
Simon Babayan
Jacqueline Badaki
Roberto Badaró
Jonathan Baell
Karoun Bagamian
Rebecca Baggaley
Lilian Bahia-Oliveira
Maria Bahia
Robin Bailey
J. Baird
Stephen Baker
Nandhakumar Balakrishnan
Rafael Balana Fouce
Oliver Balmer
Gregory Bancroft
Claudio Bandi
Gad Baneth
James Bangs
Melanie Bannister-Tyrrell
Ashley Banyard
Changjun Bao
Clara Barbiéri
Susana Barbosa
Corentin Barbu
Christovam Barcellos
Beatrice Barda
Maria Bargues

Christopher Barker
Celine Barnadas
Tamsin Barnes
Yves Barogui
Jean Carlos Barrado
Manoel Barral-Netto
ME Barrandeguy
Jacques Barrat
Roberto Barrera
Alan Barrett
Kim Barrett
Michael Barrett
Alyssa Barry
Daniella Bartholomeu
David Barton
Joziana Barçante
Miguel Basombrio
Quique Bassat
Anirban Basu
Soumyava Basu
Paul Bates
Roger Bate
Katherine Battle
Chris Bayne
Ifor Beacham
Sílvia Beato
Coreen Beaumier
Baltazar Becerril
Jeffrey Becker
Robin Beech
Susanta Behura
Marcus Beissner
Eric Beitz
Frederick Beland
Lesley Bell-Sakyi
Steve Bellan
David Bell
Steven R. Belmain
Mark Benbow
Jason Bennett
Shannon Bennett
Sasisekhar Bennuru
Joshua Benoit
Zvi Bentwich
Staffan Bergström
Luiz Bermudez
Kristen Bernard
Caryn Bern
Matthew Berriman
Andrea Berry
Paul Bessell
Kovi Bessoff
Bernard Bett
Stephen Beverley
Sarah Bevins
Fernando Bezerra
Ashish Bhalla
Sudha Bhattacharya
Sujit Bhattacharya
Purva Bhatter
Rudra Bhowmick
Adisak Bhumiratana
Gouri Sankar Bhunia
Suzanne Bialek-Davenet
Ralf Bialek
Tihana Bicanic
Quentin Bickle
Helle Bielefeldt-Ohmann
Franziska Bieri
Karolien Biesheuvel-Leliefeld
Abigail Bigham
Mirna Biglione
Fred Binka
Bryce Binstadt
Brian Bird
Nana Biritwum
David Bishai
William Bishai
Zeno Bisoffi
Sylvie Bisser
Jason Blackburn
Stuart Blacksell
Jenefer Blackwell
William Black

Carol Blair
Lynsey Blair
Isobel Blake
Nicholas Bland
Lucas Blanton
James Bliska
Bradley Blitvich
Maria Heloísa Blotta
Lucille Blumberg
Seth Blumberg
Daniel Boakye
Boakye Boatin
Moses Bockarie
Helge Bode
Hurrem Bodur
Paolo Boffetta
Christian Bogdan
Andrea Boggild
Mariana Boité
Natalia Bolaños
Wagner Bonat
Matthew Bonds
Bassirou Bonfoh
Mariangela Bonizzoni
Maciej Boni
Ken Boorom
Alison Booth
Gadi Borkow
Monica Borucki
Silvia Boscardin
Maria Laura Boschiroli
Christopher Bosio
Kwabena Bosompem
Monica Botelho
Emmanuel Bottieau
Carezza Botto-Mahan
Christian Bottomley
Gaelle Boulet
Aida Bouratbine
Pascale Bourhy
Claire Bourke
Kostas Bourtzis
Donald Bouyer
Jérémy Bouyer
Maha Bouzid
Richard Bowen
Terry Bowlin
Dwight Bowman
Alexis Boyd
Zbynek Bozdech
Doug Brackney
Timothy Bradley
Cynthia Braga
Ekanem Braide
Allan Brasier
Martin Bratschi
Aaron Brault
Francisco Bravo
Kelly Brayton
Reginaldo Brazil
Klaus Brehm
Minka Breloer
Simone Frédérique Brenière
David Brett-Major
Paul Brett
Luis Brieba
Deborah Briggs
Robert Brinkerhoff
Seth Britch
Carlos Brito
Cristiana Brito
Warwick Britton
Constança Britto
Olivier Briët
Christopher Broder
Cláudia Brodskyn
Simon Brooker
Basil Brooke
Russell Brooke
Elizabeth Brouwer
Jeremy Brownlie
John Brownstein
Corrie Brown
Dennis Brown

Hannah Brown
Lucy Brown
Wendy Brown
Oscar Bruna-Romero
Enrico Brunetti
Luc Brun
Laurent Brutus
Jose Bubis
Bruno Bucheton
Philippe Buchy
Gregory Buck
Philip Budge
Christine M. Budke
Pierre Buekens
Lilian Bueno
Hugo Bugoro
Alexander Bukreyev
Donald Bundy
Dora Buonfrate
Stewart Burgess
Mark Burgman
Thomas Burkott
Barbara Burleigh
Alejandro Buschiazzo
Juan Bustamante
Vicki Butenschoen
Alison Buttenheim
Laurence Buxbaum
Peter Bütikofer
Ramachandran C P
Miguel Cabada
Pedro Cabrales
Gonzalo Cabrera
Adalgisa Caccone
Conor Caffrey
Damien Caillaud
Jianping Cai Cai
Claudia Calegaro-Marques
Vera Calich
Guy Caljon
Juan Calvete
Vitaliano Cama
Caroline Cameron
Lindsay Campbell
Antonio Carlos Campos de Carvalho
Ricardo Campos
Renata Candido
Anthony Cannella
Luz Cano
Cinzia Cantacessi
Van-Mai Cao-Lormeau
Beniamino Caputo
Hélène Carabin
Eric Cardinale
Marta Cardinal
Mariana Cardoso
Yves Carlier
Clotilde Carlow
Elizabeth Carlton
Jane Carlton
Mariângela Carneiro
Jason Carnes
Hernán Carrasco
Christine Carrington
Mark Carrington
Juan Carrique-Mas
Jay Carroll
K. C. Carter
Nicola Carter
William Carter
Lucas Carvalho
Marilia Carvalho
Tecia Carvalho
Claudio Casanova
Jorge Casseb
Maria Cassera
Léa Castellucci
Maria Castillo
Helena Castro
Marcia Castro
Flaminia Catteruccia
Luciano Cavalcanti
Marta Cavalcanti

Juan Manuel Fernández Cean
Giuliano Cecchi
Maria Cecere
Carla Cerami
Laura Cervi
Marty Cetron
Dave Chadee
jong-Yil Chai
Frédérique Chammartin
Donald Champagne
Kwang-Poo Chang
Yung-Fu Chang
Marion Chan
Dennis Chao
François Chappuis
Theeraphap Chareonviriyaphap
Remi Charrel
Mitali Chatterjee
Gautam Chaudhuri
Luis Fernando Chaves
Ian Cheeseman
Wei Cheng
Wei-June Chen
Ya-Lei Chen
Véronique Chevalier
Roma Chilengi
Neil Chilton
Margaret Chinbuah
Sadegh Chinikar
Nakul Chitnis
Lars Chittka
Charles Chiu
Fidelis Cho-Ngwa
Min-Ho Choi
Bruno Chomel
Olivier Chosidow
Valerie Choumet
Anuradha Chowdhary
Yen-Hung Chow
Jean-Paul Chretien
Rob Christley
Ana Chudzinski tavassi
Justin Jang Hann Chu
Donato Cioli
Marc Ciosi
Patricia Cisalpino
Hannah Clapham
Chris Clarkson
Clifford Clark
Gary Clark
Christine Clayton
Sarah Cleaveland
John Clemens
Archie Clements
Gilles Clermont
Aurelie Cobat
Claudia Codeço
Giovanini Coelho
Paulo Coelho
Theresa Coetzer
Lark Coffey
Lee Cohnstaedt
Paul Coleman
Frank Collins
Gonzalo Colmenarejo
Felipe Colón-González
Inaki Comas
Fatima Conceição-Silva
Ulisses Confalonieri
Robert Cooper
Anabela Cordeiro-da-Silva
Artur Cordeiro
Marli Cordeiro
Rossana Cordeiro
S. Cornelie
Muriel Cornet
Giampietro Corradin
Dalmo Correia
Andrew Corwin
Carlo Costantini
Carlos Costa
Jane Costa
Gláucia Cota

Peggy Cotter
Thérèse Couderc
Jean Coulibaly
Pierre Couppié
Jannelle Couret
Orin Courtenay
Fabrice Courtin
Iliano Coutinho-Abreu
Robson Coutinho-Silva
Jonathan Cox
Christina Coyle
Philip Craig
Allan Cripps
Charles Criscione
Julio Croda
Simon Croft
Peter Crompton
Elizabeth Cromwell
George Cross
Robert Cross
Gregory Crowther
John Crump
Jorge Cruz-Reyes
Angela Cruz
Israel Cruz
Paul Cullen
Richard Culleton
David Cummiing
Scott Cummins
Edecio Cunha-Neto
Charles Cunningham
Elisa Cupolillo
Ed Cupp
Jeffrey Currier
Bart Currie
Jason Curtis
Stephen Cusack
Sally Cutler
Krystyna Cwiklinski
Valerie D'Acremont
Kounbobr Roch DABIRE
Hélène DELATTE
Alda Maria DaCruz
Renato DaMatta
Yankum Dadzie
John Dalton
David Dance
Eric Dannaoui
Alistair Darby
Marie-Laure Dardé
Richard Dart
Kali Das
Murari Das
Pradeep Das
Kashmira Date
Claudia Daubenberger
Gail Davey
Dan David
John David
Shireen Davies
Stephen Davies
Stephanie Davis
Emily Dawson
Egon Daxbacher
Gustavo Dayan
Nicholas Day
Tim Day
Katinka De Balogh
Marina De Carli
Solange De Castro
Harry De Koning
Thierry De Meeus
Ricardo DeMarco
Matthew Dean
Stijn Deborggraeve
Alain Debrabant
Saskia Decuypere
Abraham Degarege
Kevin Deitz
Oscar Del Brutto
Frederic Delbac
Peter Delputte
Eric Delwart
Caroline Demangel

Ana Denicola
Lindsay Dent
Raffy Deray
Kebede Deribe
Albert Descoteaux
David Deshazer
Mahalia Desruisseaux
Alain Dessein
Raul Destura
Amélie Desvars
Eileen Devaney
Gregor Devine
Ranadhir Dey
Mawlouth Diallo
Michael Diamond
Rosario Diaz Gonzalez
David Diemert
Ralf Dietzgen
Peter Diggle
George Dimopoulos
Rhoel Dinglasan
Lileia Diotaiuti
Ermias Diro
Colette Dissous
Katharina Dittmar
Dirk Dittmer
Stephen Dobson
Roberto Docampo
Hazel Dockrell
Mike Doenhoff
Olga Dolnik
Stefan Dongus
Martin Donnelly
Sheila Donnelly
Helen Donoghue
Robert Don
Pierre Dorny
Patricia Dorn
Alberto Juan Dorta-Contreras
Seydou Doumbia
Kimberly Dowd
Jennifer Downs
Steven Dow
Michel Drancourt
Michael Drebot
Nicole Driessen
Guangcai Duan
Geraldo Duarte
JP Dubey
Anuradha Dube
Jean-Bernard Duchemin
Jean-Claude Dujardin
J. Stephen Dumler
Veasna Duong
Kendra Dupuy
Ravi Durvasula
Sandra Durães
Michael Duszenko
Malcolm Duthie
Walderez Dutra
Shanta Dutta
Sujoy Dutta
Gérard Duvallet
Jan Dvorak
Vit Dvorak
Salome Dürr
Praphathip Eamsobhana
Julian Eaton
Gregory Ebel
Mark Eberhard
Hideki Ebihara
Michael Eddleston
Miriam Eddyani
Robert Edelman
Tansy Edwards
Daniel Eibach
Lars Eisen
Rebecca Eisen
Uwem Ekpo
Hail El-Abdel
Maria Carolina Elias
Richard Elliott
Brett Ellis
Magda Ellis

Jerrold Ellner
Dia-Eldin Elnaiem
Paul Emerson
David Engman
Markus Engstler
Joerg Epplen
Scott Erdman
Marina Eremeeva
Bobbie Erickson
Klaus Ersfeld
Ananias Escalante
Alejandro Escobar-Gutiérrez
Sergio Espinola
Bertha Espinoza
Ana Espino
Maria Cristina Espírito-Santo
José Esteban Sanchis
Mariano Esteban
J Esterhuizen
Philippe Esterre
Magnus Evander
Brian Evans
Holly Evans
Amara Ezeamama
Pierre-Edouard FOURNIER
Milosz Faber
Ahmed Fahal
Anna-Bella Failloux
Rick Fairhurst
Jessica Fairley
Walter Fairlie
Michaela Fakiola
Franco Falcone
Padraic Fallon
Ferric Fang
Liqun Fang
Rong Fang
Chia Fan
Hui Wen Fan
Mauricio Farfan
Andrew Farlow
Nicolas Fasel
Quek Fatt
Dunia Faulx
Vinicius Fava
Silvana Regina Favoretto
Ousmane Faye
Christine Fehlner-Gardiner
Heinz Feldmann
Ingrid Felger
Lukas Fenner
Ana Paula Fernandes
Ana Fernandez-Sesma
Stefan Fernandez
LakKumar Fernando
Carmen Fernández-Becerra
Matthew (Matt) Ferrari
Gonçalo Ferraz
Elizabeth Ferreira
Marcelo Ferreira
Martin Ferris
Elisabeth Fichet-Calvet
Mark Field
Joshua Fierer
Juliana Fietto
Luisa Figueiredo
Peter Figueroa
Katja Fink
Russell Finley Jr.
Robert Finn
Anthony Fiore
Pier Luigi Fiori
Alireza Firooz
Egil Fischer
Marc Fischer
Peter Fischer
Susan Fisher-Hoch
Christopher Fitzpatrick
J. Bert Flanegan
Abraham Flaxman
Lawrence Fleckenstein
Bernhard Fleischer
Fiona Fleming
Agnes Fleury

Laurence Flevaud
Michael Flint
Ana Flisser
Gordon Florence
Adriana Flores
Robin Flynn
Dina Fonseca
Simone Fonseca
Anthony Fooks
Naomi Forrester
Brett Forshey
Guillaume Fournié
Rob Fowler
Annette Fox
Ellen Fox
Jay Fox
Donald Francis
Stephen Francis
Jose Franco
Jose Ramon Franco
Nikolaos Frangogiannis
Alexander Franz
Malcolm Fraser
David Freedman
Matthew Freeman
Hector Freilij
Dayvison Freitas
Michael French
Manuel Fresno
Ute Frevert
Jennifer Friedman
Michal Fried
Ilya Frolov
Bastian Fromm
Ines Fronteira
Hans-Peter Fuehrer
Agustin Fuentes
Ricardo Fujiwara
Douglas Fuller
Sebastian Funk
Simon Funnell
Zhen F. Fu
Statisticians GWU
Albis-Francesco Gabrielli
Sarah Gabriel
Holly Gaff
Maria Clara Galhardo
Mary Galinski
Renee Galloway
Stephen Gallo
Manoj Gambhir
Jean-Pierre Gangneux
Sandipan Ganguly
Lisa Ganley-Leal
Sreenivas Gannavaram
Teresa Garate
Amadou Garba
Andres Garchitorena
Hector Garcia
Aysen Gargili
Nisha Garg
Robin Gasser
Luca Gattinoni
Michael Gaunt
Bruce Gaynor
Soraya Gaze
Pedro Gazzinelli-Guimarães
Ricardo Gazzinelli
Timothy Geary
Christine Gee
Florian Gehre
Lee Gehrke
Brian Geiss
Huub Gelderblom
Peter Geldhof
Annemieke Geluk
Fernando Genta
Marta Maria Geraldes Teixeira
Antoine Gessain
Arthur Getis
Elodie Ghedin
Anirban Ghosh
Lorenzo Giacani
Paul Giacomin

Katherine Gibson-Corley
Wendy Gibson
Charity Gichuki
Jeremy Gilbreath
Carol Gilchrist
Jeremie Gilles
Robert Gilman
Robert Gilmore
Horacio Gil
Natasha Girgis
Núria Gironès
Rodolfo Giunchetti
Ippolito Giuseppe
Gregory Glass
Cyrille Goarant
Geoffrey Gobert
Anna Goldberg
Tony Goldberg
Nick Golding
Kenneth Gollob
Bruno Gomes
Karina Gomez
Ricardo Gomez
Jie Gong
Nelder Gontijo
Angel Gonzalez
Armando Gonzalez
Dolores González-Pacanowska
David Goodman
Pamina Gorbach
Aubree Gordon
David Gordon
David Gorla
Hiro Goto
Nicole Gottdenker
Bruno Gottstein
Dimitrios Gouglas
Ernest Gould
Sebastien Gourbiere
Brian Gowen
Charlotte Gower
Luigi Gradoni
Carlos Graeff-Teixeira
Joel Graff
Marina Gramiccia
Patricia Graves
Stephen Graves
David Greenberg
Robert Greenberg
Bryan Greenhouse
Sharone Green
Aric Gregson
Christoph Grevelding
Emily Griffiths
Maria Eugenia Grillet
Edmundo Grisard
Martin Grobusch
Jacques Grosset
Christoph Grunau
Duane Gubler
Paulo Guedes
Pascale Gueirard
Humberto Guerra
Claudia Guezala
Felipe Guhl Nannetti
Rhainer Guillermo-Ferreira
Hélène Guis
Abba Gumel
Sharmini Gunawardena
Rajni Gunnala
Shivali Gupta
David Gurarie
Rodrigo Gurgel-Gonçalves
Maria Lucia Guther
Gamaliel Gutierrez
José Gutiérrez
Julie Gutman
Bruno Guy
Theresa Gyorkos
Miklós Gyuranecz
Ricardo Gürtler
Eveline Hürlimann
Sung-Tae HONG

David Haake
Jurgen Haanstra
Abdulrazaq Habib
Andrew Haddow
Julius Clemence Hafalla
Ferry Hagen
Amy Hagopian
Rosane Hahn
Asrat Hailu
Stephen Hajduk
Ramin Hakami
David Hallengärd
Jo Halliday
Roy Hall
Scott Halstead
Kate Halton
Omar Hamarsheh
Frederik Hammes
Katie Hampson
Sukwan Handali
Patrick Hanington
Kathryn Hanley
Laura Harburger
Richard Hardy
Edward Harhaj
David Harley
Billy Harnett
Robert Harrison
Eva Harris
Jason Harris
Shimon Harrus
Susanne Hartmann
Amy Hartman
Rudy Hartskeerl
Marina Harvie
Futoshi Hasebe
Epco Hasker
Jan Hattendorf
Susanna Hausmann-Muela
John Hawdon
Thomas Hawn
David Hayman
Roderick Hay
David Heath
Bethany Hedt-Gauthier
Norbert Heinrich
Mark Heise
Ryan Hemme
Andrea Henriques-Pons
Debroski Herbert
Vincent Herbreteau
Melinda Hergert
Merce Herrero
Jana Hertel
Jorg Heukelbach
James Hewitson
Roger Hewson
Peter Heydemann
Martin Hibberd
Kazuya Hidari
Julian Hillyer
Jay Hinton
Kenji Hirayama
Alec Hirsch
Natasha Hochberg
Ary Hoffmann
James Hogle
Cornelis Hokke
Lindy Holden-Dye
Celia Holland
T Deirdre Hollingsworth
Edward Holmes
Kathryn Holt
Akira Homma
Robert Hondal
Sung-Jong Hong
Anna Honko
Robert Hontz
D. Craig Hooper
Pamela Hooper
Adrian Hopkins
Ricardo Hora
Michael Horseman
Olaf Horstick

John Horton
Paul Hoskisson
Duane Hospenthal
Parviez Hosseini
Peter Hotez
Jos Houdijk
Eric Houpt
Rosalind Howes
Paulo Ho
Michael Hsieh
Huan Huang
Grant Hughes
Leon Hugo
Mahmoud Huleihel
Debbie Humphries
Judith Humphries
Lauren Hund
Elizabeth Hunsperger
Jae-Seoun Hur
Kris Huygen
Kate Huyvaert
Nguyen Huy
Rongliang Hu
Jimee Hwang
Stephen Hyslop
Alexander Idnurm
Ikuo Igarashi
Allison Imrie
Hyunsoon Im
Daniel Inaoka
Satoshi Inoue
Geoffrey Isbister
Krishna Isloor
Akira Ito
Louise Ivers
Shuji Izumo
Abdul Jabbar
Andrew Jackson
Joseph Jackson
Ronald Jackson
Yves Jackson
Julie Jacobson
Thomas Jaenisch
Pooja Jain
Ronan Jambou
Vincent Jamonneau
Michel Jancloes
Dragana Jankovic
Ana Jansen
Armando Jardim
Edward Jarroll
Emilie Javelle
Claire Jenkins
David Jenkins
Emily Jenkins
Helen Jenkins
Grant Jenkin
Jan Jensen
Aaron Jex
Tie-Wu Jia
Maria Isabel Jimenez
Xia Jin
Maria Johansen
Michael Johansson
Paul Johnson
Annamma John
Christopher Jones
Douglas Jones
Kathryn Jones
Malcolm Jones
Therésa Jones
Somchai Jongwutiwes
Peter Jourdan
Justin Julander
Steven Juliano
Thomas Junghanss
Sushil Kabra
Malika Kachani
Rebekah Kading
Barbara Kahl
Stuart Kahn
Adriana Kajon
Siripen Kalayanarooj
Joan Kalyango

Joseph Kamgno
Osamu Kaneko
Gagandeep Kang
Biao Kan
Ray Kaplan
P Karani
Timothy Karr
Shinji Kasai
Fatah Kashanchi
Joldoshbek Kasymbekov
Moses Katabarwa
Rebecca Katz
Jim Kaufman
Deepak Kaushal
Nutan Kaushik
Hitoshi Kawada
Paul Kaye
Mirdad Kazanji
Rudovick Kazwala
Jeremy Keenan
Kylene Kehn-Hall
Bart Keijser
Jennifer Keiser
Louise Kelly-Hope
John Kelly
Maggi Kelly
Steven Kelly
Volkhard Kempf
Bernard Keraita
Peter Kern
Ali Khamesipour
Asif Khan
Imtiaz Khan
Shahid Khan
Mustafa Khokha
Kaveh Khoshnood
Jimmy Kihara
Jun Kikuchi
Gerry Killeen
Peter Kima
Jessica Kim
Kwang Kim
Young Eun Kim
Charles King
Jonathan King
Craig Kinnear
Safari Kinung'hi
Amy Kirby
Louis Kirchhoff
Theo Kirkland
Beth Kirkpatrick
Martyn Kirk
Kiyoshi Kita
Sonia Kjos
Sabra Klein
Amy Klion
Karl Klose
Stephen Klotz
Livia Kmetzsch
Kayla Knilans
Charles Knirsch
Darryn Knobel
Stefanie Knopp
Dave Knox
David Knox
Giselle Knudsen
Gary Kobinger
Oliver Koch
Richard Kock
Jacob Koella
Cristian Koepfli
Alphonsine Koffi
Mathurin Koffi
Edward Kohi
Alain Kohl
Nikolay Kolev
Martin Kollman
Anne-Brit Kolsto
Yoon Kong
Bart Kooi
Steven Kopp
Joseph Koroma
Poonum Korpe
Michael Kosoy

Ivanete Kotait
Karen Kotloff
Michalis Kotsyfakis
Andrew Kotze
Artemis Koukounari
Daniella Kovacsics
Sari Kovats
Uriel Koziol
Jürgen Krücken
Elliot Krafsur
Thomas Kreil
Natacha Kremer
Alison Krentel
Marco Krieger
Manoj Krishnan
Igor Krizaj
Pascale Kropf
Claire Kubelka
Mark Kuhlenschmidt
Richard Kuhn
Daniel Kulke
Martin Kulldorff
NP Kuman
Sanjay Kumar
Armand Kuris
Jonathan Kurtis
John Kusel
Ivan Kuzmin
Eliningaya Kweka
Babu L. Tekwani
Anne Camille La Flamme
A. Desiree LaBeaud
James LaCourse
Nicole LaRonde-LeBlanc
Michaela Lackner
Monique Lafon
Xavier Lamballerie
Poppy Lamberton
Louis Lambrechts
Patrick Lammie
Tommy Tsan-Yuk Lam
Claudio Lanata
Roberto Lande
Jordi Landier
Ingeborg Langohr
Gordon Langsley
Emily Lankau
Felix Lankester
Joseli Lannes-Vieira
Carlos Lanusse
Humberto Lanz-Mendoza
Gregory Lanzaro
Dhafer Laouini
Renee Larocque
Edmundo Larrieu
Sébastien Larréché
Susana Laucella
Larry Laughlin
Colleen Lau
Rachel Lawrence
Laura Layland
Claudio Lazzari
Stephen Leak
Fabio Leal
Marc Lecuit
Nathan Ledeboer
Rosemary Lees
Johannis Leeuwenburg
Bruce Lee
Min-Shi Lee
Rogan Lee
Yoosook Lee
Mathieu Legros
Mariana Leguia
Maria Leguizamon
Iuri Leite
David Leitsch
Laura Leiva
Veerle Lejon
Maud Lelu
Tiziana Lembo
Audrey Lenhart
Adrijana Leonardi

Elli Leontsini
Guillermo Leon
Yee-Sin Leo
Ganjana Lertmemongkolchai
Andres Lescano
Justin Lessler
Daniel Leung
Bruno Levecke
Paul Levett
Ann Levin
Karen Levy
Michael Levy
Michael Lewis
Song Liang
Yousheng Liang
Michael Libman
Daniel Libraty
Tore Lier
Bernhard Liese
Thomas Lietman
Direk Limmathurosakul
Poh-Lian Lim
José Angelo Lindoso
Steven Lindsay
Mark Lindsley
Jo Lines
Vishwanath Lingappa
Hualiang Lin
Yi-Ling Lin
Peter Lipke
Florigio Lista
Bin Liu
Qiyong Liu
Shan-Lu Liu
Shu-Lin Liu
Cheng-Yi Li
Ya-Pin Li
Alejandro Llanos
Martin Llewellyn
Alun Lloyd
Giovanni Lo Iacono
Philip LoVerde
Mario Lobigs
Eric Loker
Bruno Lomonte
HOANG Truong Long
Kanya Long
Maureen Long
Thavy Long
Andreas Lopata
Anna Lena Lopez
Manuel Lopez
Marcelo Lorenzo
Olivier Lortholary
Hannelore Lotter
Hechmi Louzir
Rachel Lowe
Jenny Low
Daniel Franz Lozano Beltran
Saul Lozano-Fuentes
Norma Lucena-Silva
Shirley Luckhart
Igor Lukashevich
Sheila Lukehart
Julius Lukes
Lucy Lum
Jan Lundstrom
Francisco Luquero
Alejandro Luquetti
Sara Lustigman
Pascal Lutumba
Adrian Luty
Paula Luz
Samantha Lycett
Shari Lydy
David C Lye
Rogelio López-Vélez
David Mabey
Margaret MacDonald
Calman MacLennan
Kevin Macaluso
Fabiana Machado
Rafael Maciel-de-Freitas
Charles Mackenzie

Calum Macpherson
Tariq Madani
Giordano Madeddu
Rentala Madhubala
Henry Madsen
Kalana Maduwage
Louis Maes
Ricardo Magalhaes
Stefan Magez
Jean François Magnaval
Pascal Magnussen
Ulf Magnusson
Sheryl Magzamen
Siddhartha Mahanty
Mathieu Maheu-Giroux
Renaud Mahieux
Barbara Mahon
Alexandra Mailles
Robbie Mailliard
Rick Maizels
Silas Majambere
Benjamin Makepeace
Gathsaurie Malavige
Emilio Malchiodi
Rosa Maldonado
Mwele Malecela
Wanchai Maleewong
Reza Malekzadeh
Indu Malhotra
John Malone
Rebeca Manning
Carrie Manore
Pablo Manrique Saide
Pablo Manrique-Saide
John Mansfield
Nuha Mansour
Ben Mans
Raúl Manzano Román
Chris Marcellino
Jonathan Marchant
Antonio Marcilla
Harold Margolis
Karina Mariante Monteiro
Kevin Maringer
Claudio Marinho
Silvio Mariotti
Wilfred Marissen
Lewis Markoff
Tigran Markosyan
Michael Marks
Gabriela Maron
Ernesto Marques
Joao Marques
Christina Marra
Laurent Marsollier
Denise Marston
Eric Martinez
Jose Guadalupe Martinez
Francisco Rogerlândio Martins-Melo
Coralie Martin
Diana Martin
Julio Martin
Richard Martin
Jesús Martínez-Barnetche
Andrea Marzi
Santiago Mas-Coma
Daniel Masiga
Felix Masiye
Dmitri Maslov
Robert F Massung
Alastair Matheson
Anuja Mathew
Derrick Mathias
Greg Matlashewski
Michael Matthias
Carla Mattos
Ian Maudlin
Isabel Mauricio
Patrick Mavingui
Wen Jun Ma
Charles Mbogo
Jere McBride
John McBride

Philip McCall
Christina McCarthy
James McCarthy
James McCaw
A McCollum
Glenn McConkey
Malcolm McConville
Scott McDonald
Mary Ann McDowell
Anita McElroy
Juan McEwen
Stephen McGarvey
Bradford McGwire
Jane McHowat
James McKerrow
Diane McMahon-Pratt
Don McManus
Donald McManus
W. Robert McMaster
Peter McMinn
Kirsty McPherson
Henry McSorley
Stephen McSorley
David McVey
Arianna Means
Luke Mease
Amel Meddeb-Garnaoui
Danielle Medek
Antonieta Medina Lara
Graham Medley
Jean-Louis Mege
Jane Megid
Andre Meheus
Heinz Mehlhorn
Christian Melaun
Peter Melby
Breno Mello
Cathy Mendelsohn
Shanthi Mendis
Fela Mendlovic
Daniel Mendoza
Joris Menten
Richard Merritt
Pascal Mertens
Greg Mertz
Steven Meshnick
françois-xavier Meslin
Louisa Messenger
William Messer
Jane Messina
Lynn Meurs
Dan Meyrowitsch
Isaura Meza
Paul Michels
Nicholas Midzi
Steve Mihok
Michael Miles
Javier Millan
Wolfgang Miller
Genevieve Milon
Jesus Mingorance
Gabriela Minigo
Michael Minnick
Timothy Minogue
Paola Minoprio
Eric Mintz
Democrito Miranda-Filho
Chad Mire
Oriol Mitjà
Edward Mitre
Gerald Mkoji
Mahtab Moayeri
Kayvon Modjarrad
Igor Mokrousov
Goudarz Molaei
Israel Molina
David Molyneux
Emmanuel Mongin
Severine Monnerat
Carlos Montanari
Fernando Monteiro
Silvia Montenegro
Susan Montgomery
Antonio Montresor

Lianet Monzote
Peter Moore
Sarah Moore
Andy Moorhead
Josué de Moraes
Milton Moraes
Miguel Morales
Serge Morand
Alessandra Morassutti
Melinda Moree
Silvia Moreno
David Morens
Lisa Morici
Pedro Moro
John Morrill
Ivan Morrison
Liam Morrison
Thomas Morrison
James Morris
Michelle Morters
Eric Mossel
William Moss
Claudia Motrán
Jeremy Mottram
Adrian Mountford
Angela Mousley
Catherine Moyes
Andreas Mueller
Ivo Mueller
Claire Mugasa
Samson Mukaratirwa
Swati Mukherjee
Chinmay Mukhopadhyay
Dunstan Mukoko
Grace Mulcahy
David Muller
Jason Mulvenna
Dieudonné Mumba Ngoyi
Kosta Mumcuoglu
Ulrike Munderloh
Cesar Munoz-Fontela
Claudia Munoz-Zanzi
Jose Munoz
Grace Murilla
Antonio Muro
Sean Murphy
Kristy Murray
Natasha Murray
Peter Murray
Ahmed Musa
Carla Muñoz-Antoli
Pauline Mwinzi
Charles Myers
Peter Myler
Pascal Mäser
Ingrid Müller
Mathieu Nacher
Steven Nadler
Fnu Nagajyothi
Yoshiro Nagao
Rana Nagarkatti
G Nair
Meera Nair
Hamed Najafabadi
Minoru Nakao
Hira Nakhasi
Jarlath Nally
Pejman Naraghi-Arani
Kanwar Narain
Sukanya Narasimhan
Takeshi Nara
Bakela Nare
Eduardo Nascimento
Roger Nasci
Theodore Nash
Krishnamurthy Natarajan
Uma Chandra Mouli Natchu
Michael Nathan
Indira Nath
Fernando Navarro-Garcia
Yukifumi Nawa
Monica Nayakwadi Singer
Martial Ndeffo Mbah
Paul Ndegwa

Deborah Negrão-Corrêa
P Nejsum
Susana Nery
Andreas Neumayr
Anna Newton
Paul Newton
Samuel Newton
Charles Nfon
Jeremiah Ngondi
Paul Nguewa
Kieu Anh Nguyen
Lisa Ng
William Nicholson
Elizabeth Nichols
Mark Nichter
Mark Nicol
Matthias Niedrig
Alex Nielsen
Morten Nielsen
William Nierman
Birgit Nikolay
Eric Nilles
Alasdair Nisbet
M. Njenga
Flobert Njiokou
Céline Nkenfou
Aline Nobre
Ane Nodtvedt
Melissa Nolan
Tony Nolan
Neil Norcross
Rusli Nordin
Fernando Noriega
Paul Norman
Douglas Norris
Florian Noulin
Norbert Nowotny
Harry Noyes
Issarang Nuchprayoon
Jack Nunberg
Thomas Nutman
Patricia Nuttall
Dougbeh-Chris Nyan
Susanne Nylen
Daniel O Brien
Carolyn O'Brien
Roberta O'Connor
Vincent O'Connor
Eamon O'Dea
Kaitlin O'Keefe
Seth O'Neal
Hester O'Neill
Sandra O'Neill
Richard Oberhelman
Brigit Obrist
Clara Ocampo
Maria Ochoa
Theresa Ochoa
Torsten Ochsenreiter
Christian Ockenhouse
Peter Odermatt
Nicholas Ogden
Tinuade Ogunlesi
Nobuo Ohta
Kazunori Oishi
Kenichi Okamoto
Joseph Okeibunor
Anna Okello
Chukwu Okoronkwo
Chinyere Okoro
G. Richard Olds
Clelia Oliva
Fabiano Oliveira
Guilherme Oliveira
Annette Olsen
Judy Omumbo
Ambrose Onapa
Mario Ono
Eng Eong Ooi
Anna Oommen
Kenneth Opara
Guadalupe Ortega-Pierres
Yoshio Osada
Mike Osei-Atweneboana

Agneta Oskarsson
William Oswald
Domenico Otranto
Eric Ottesen
Marc Ouellette
Johnson Ouma
Helen Owen
Richard Oxborough
Christopher M PARRY
SANTHOSH PUTHIYAKUNNON
M. Andreína Pacheco
Christopher Paddock
Slobodan Paessler
Wendy Page
Sithithaworn Paiboon
Vivek Pai
Ricardo Palacios
Søren Paludan
Subhamoy Pal
Utpal Pal
Bo Pang
S.P Pani
Ming-Jeng Panmjpan@ctust.edu.tw
Jingcao Pan
Weiqing Pan
F. Nina Papavasiliou
Raul Pardo
David Parenti
Manmohan Parida
Daniel Paris
Aristeidis Parmakelis
Isabel Parraga
Parviz Parvizi
Mercedes Pascual
Macej Pastuszczak
Fabiana Paula
Ross Paveley
George Pavlakis
Janusz Paweska
Terry Pearson
Jorge Pedrosa
Rosanna Peeling
Herve Pelloux
Marcia Pereira de Oliveira
Mercio PereiraPerrin
Claudio Pereira
Iris Pereira
Marcos Pereira
David Perera
Rushika Perera
Ana Perez
Maria Periago
Aditya Perkasa
Alex Perkins
Pascale Pescher
Felipe Pessoa
Christine Petersen
Eskild Petersen
A. Townsend Peterson
Nathan Peters
William Petri, Jr.
Kenneth Pfarr
Mavinga Delphin Phanzu
Mario Philipp
Richard Phillips
Antoinette Piaggio
Mathieu Picardeau
Stéphane Picot
Raymond Pierce
Ted Pierson
David Pigott
Pavitra Pillay
Benjamin Pinsky
Eduardo Pinto
Sébastien Pion
R. Pitts
Virginia Pitzer
Juan Pizarro
Alexander Pletnev
Michael Pollastri
Anastasia Polycarpou
Betty Poole-Smith
Stephen Popper

Travis Porco
Kevin Porter
Miriam Postan
Jan Potempa
Rodolphe Poupardin
Ann Powers
Nicolas Praet
Manu Prakash
Clarissa Prazeres da Costa
Diego Preciado
Ric Price
Jeffrey Priest
Eric Prina
Gerardo Priotto
Anna Protasio
Michael Prouty
Carla Pruzzo
Rachel Pullan
Arto Pulliainen
Narain Punjabi
Chaturong Putaporntip
Firdausi Qadri
Cheng-Feng Qin
Thomas Quack
Flávio Queiroz-Telles
Rupert Quinnell
Juliana Quintero
Wagner Quintilio
Jose REQUENA
RODRIGO RIBEIRO-RODRIGUES
Maia Rabaa
Ana Rabello
Jorge Rabinovich
Geert Raes
Sima Rafati
Vedantam Rajshekhar
Kapa Ramaiah
Marcelo Ramalho-Ortigao
Rajendranath Ramasawmy
Janine Ramsey
Shoba Ranganathan
Didier Raoult
Mrinalini Rao
Jayne Raper
Chad Rappleye
Guilhem Rascalou
Gordana Rasic
David Rasko
David Rasmussen
Thomas Rasmussen
Kavi Ratanabanangkoon
Ruwan Ratnayake
Jean Baptiste Rayaisse
Julian Rayner
Paul Ready
Maria Rebollo Polo
Maria Rebollo
Douglas Reed
Steven Reed
Ryan Rego
Simon Reid
Lisa Reimer
Neil Reiner
Robert Reiner
Carolina Reisenman
William Reisen
Michael Reiskind
Alexandre Reis
Bernardo Reis
Paul Reiter
Richard Reithinger
Jan Remme
Alfons Renz
Angela Restrepo
Arturo Reyes-Sandoval
Todd Reynolds
Antonio Ribeiro
Claudio Tadeu Daniel Ribeiro
José Ribeiro
Paula Ribeiro
Monica Richardson
Allen Richards
Frank Richards

S Richards
Jan Richardus
Rebeca Rico-Hesse
Michael Riehle
Paula Rigato
Leen Rigouts
Gabriel Rinaldi
Laura Rinaldi
Dagmar Ringe
Maria Rios
Rita Rio
Scott Ritchie
Koert Ritmeijer
Uwe Ritter
Windell Rivera
Ana Rivero
Carlos Robello
Chrissy Roberts
Derrick Robinson
Mark Robinson
Richard Robison
Manoel Otávio Rocha
Leda Roche
Ilia Rochlin
Joacim Rocklöv
Barry Rockx
Jean Rodgers
Louise Rodino-Klapac
Isabel Roditi
Chathuraka Rodrigo
Fernando Rodrigues
Mauricio Rodrigues
Isabel Rodriguez Barraquer
Mario Rodriguez-Perez
Dawn Roellig
Amira Roess
Ester Roffe
Michael Rogan
Stephen Rogerson
David Rogers
Matthew Rogers
Petra Rohrbach
Bernard Roizman
Mirko Rojas Cortez
Antonieta Rojas de Arias
William Roldán
David Rollinson
Gustavo Romero
Thomas Romig
Phyllis Romijn
Catherine Ronet
Pornpimol Rongnoparut
Pierre Roques
Rick Rosatte
Kerstin Rosenberger
Helene Rosenberg
Benjamin Rosenthal
Mara Cecilia Rosenzvit
Carla Rothlin
Alan Rothman
Emmanuel Roux
Edward Rowan
David Rowlands
Craig Roy
S Roy
Syamal Roy
Craig Ruaux
Larry Ruben
Guita Rubinsky-Elefant
Dominik Ruckerl
Robert Rudd
Gloria Rudenko
James Rudge
Jeronimo Ruiz
Silvia Runge-Ranzinger
VEDANTAM Rupa
Jonathan Rushton
Bruce Russell
Laird Ruth
Edward Ryan
Daniel Růžek
MARTHA SABOYA
Anderson Sa-Nunes
Christopher Saarnak

David Sachs
Moussa Sacko
David Sacks
David Sack
David Safronetz
Debasish Saha
Mayuko Saito
Tais Saito
Anna Maria Sales
Claudio Salgado
Padmini Salgame
Jorge Salinas
Henrik Salje
Sam Salman
Oscar Salomon
Sammy Sam-Wobo
Indira Samarawickrema
John Samuelson
James Samuel
I-Ching Sam
Ana Sanchez
Rosemary Sang
Felix Santiago
Helton Santiago
Saul Santivanez
Lucio Santos
Silvane Santos
Nancy Saravia
Fred Sarfo
Euzenir Sarno
Abhay Satoskar
Erik Sauleau
Paul Saunderson
Bernadette Saunders
Stephen Savarino
Somphou Sayasone
IC Scaletsky
Peter Schantz
Julio Scharfstein
Guenter Schaub
Esther Schelling
Christina Scherer
John Schieffelin
Alejandro Schijman
Connie Schmaljohn
Nathan Schmidt
Veronika Schmidt
Herbert Schmitz
Gabriel Schmunis
Achim Schnaufer
Jay Schneider
Maria Cristina Schneider
Frank Scholle
Gerard Schoone
Tony Schountz
Augusto Schrank
Albert Schriefer
Amy Schuh
Richard Schulz
Wolfgang Schulz
Erwin Schurr
Tom Schwan
Erich Schwarz
Herbert Schweizer
Jean-François Schémann
Gabriele Schönian
Edda Sciutto
D.M. Scollard
Tatiana Scorza
Alan Scott
Maxwell Scott
Thomas Scott
Maria Scroferneker
Carlos Seas
William Secor
Núgila Secundino
Karin Seifert
Joao Seixas
James Sejvar
Shamala Devi Sekaran
Heloisa Selistre de Araujo
Paul Selzer
Jan Semenza
Hanoch Senderowitz

julian Serrano
Carlos Sevcik
David Severson
Mohammad Shah
Alison Shakarian
Manjunath Shankar
G. Dennis Shanks
K. Krishna Sharma
Joanne Sharp
Tyler Sharp
Alex Shaw
Yujuan Shen
Mineko Shibayama
Jennifer Shield
Brian Shiels
Hiroyuki Shimizu
Ho-Joon Shin
Jyh-wei Shin
Joanna Shisler
Benyun Shi
Charles Shoemaker
Sujan Shresta
Rima Shretta
Marc Siegel
Ariel Silber
Sydnei Silva
Pere Simarro
Rachel Simmonds
Cameron Simmons
Cheolho Sim
Benson Singa
Balbir Singh
Neeloo Singh
Sarman Singh
Steven Sinkins
Photini Sinnis
Jair Siqueira-Neto
André Siqueira
Paiboon Sithithaworn
Philip Skuce
Rachel Slayton
Michel Slotman
Scott Small
Darci Smith
David Smith
Duncan Smith
Terry Smith
Henk Smits
Jolanda Smit
Michael Smout
Georges Snounou
Milena Soares
Rodrigo Soares
María Eugenia Socías
Maria de Nazaré Soeiro
Laia Solano-Gallego
Philippe Solano
Aldo Solari
Dominique Soldati-Favre
ME Soliman
Anthony Solomon
Pradya Somboon
Yajun Song
Lynn Soong
Ghislain Sopoh
Vincent Soriano
Frank Sorvillo
Sergio Sosa-Estani
Ketty Soteriadou
Marc Souris
Gerald Spaeth
Robert Spear
Sabine Specht
Benjamin Speich
John Spencer
Gilberto Sperandio da Silva
Niko Speybroeck
Paul Spicer
Stephen Spitalnik
Gary Splitter
Dave Spratt
David Spray
Anon Srikiatkhachorn
Banchob Sripa

Ashley St John
Miguel Stadecker
Simona Stager
Hans-Christian Stahl
Claire Standley
Michelle Stanton
Eike Steinmann
Peter Steinmann
Ivo Steinmetz
John Stenos
Henry Stephens
Andy Stergachis
Jeremy Sternberg
Sharon Stevelink
Brian Stevenson
Lori Stevens
Aisha Stewart
Philip Stewart
Ymkje Stienstra
Eileen Stillwaggon
O. Stine
Steven Stoddard
Wilma Stolk
Bob Storey
J. Russell Stothard
Daniel Streblow
Daniel Streicker
Adrian Streit
Thomas Strickland
Daniel Strickman
Clare Strode
Christina Strube
Claudio Struchiner
Brian Suarez Mantilla
Balanehru Subramanian
Sebastian Suerbaum
Hiromu Sugiyama
Andreas Suhrbier
William Sullivan Jr.
David Sullivan, Jr.
Yi-Cheng Sun
Mehul Suthar
Colin Sutherland
Sutas Suttiprapa
Takashi Suzuki
Staffan Svard
Paul Swoboda
Sharifah Faridah Syed Omar
Zainulabeuddin Syed
Marcelo Sztein
Fabienne Tachini-Cottier
Wagner Tafuri
Ayato Takada
Ratree Takhampunya
Ernest Tambo
Paul Tambyah
Aparecida Tanaka
Nelson Tang
Qing Tang
Herbert Tanowitz
Cindy Tan
Feng Tan
Dingyin Tao
Mushfiqur Tarafder
Rick Tarleton
Wagner Tassinari
Carla Taylor
Erik Taylor
Hugh Taylor
Mark Taylor
Martin Taylor
Matthew Taylor
Andrea Teixeira-Carvalho
Antonio Teixeira
Maria Glória Teixeira
Mauro Teixeira
Santuza Teixeira
Afework Tekle
Michael Templeton
Sharon Tennant
Luis Terrazas
Marcel Teunissen
Saravanan Thangamani
Oriel Thekisoe

Megan Thoemmes
Lian Thomas
Matthew Thomas
Stephen Thomas
Ricardo Thompson
Nicholas Thomson
Kirkby Tickell Tickell
Aloisius Tielens
Alexandre Tiendrebeogo
Laura Tipton
Inaki Tirados
Anna Tischler
Daniel Tisch
Angela Tobón
Jim Todd
Zilahatou Tohon
Kay Tomashek
Oyewale Tomori
Ana Tomás
Shilu Tong
Noel Tordo
Paul Torgerson
Conchita Toriello
Jeffrey Tornheim
Steve Torr
José Tort
Yves Tourre
Jonathan Towner
Yara Traub-Csekö
Bruno Travi
Omar Triana-Chávez
Lucienne Tritten
Francois Trottein
A Trudgett
Richard Truman
Carine Truyens
Jih-Jin Tsai
Renée M. Tsolis
Ataru Tsuzuki
Apichai Tuanyok
Michael Turelli
Michael Turell
Hugo Turner
Joseph Turner
Kevin Tyler
María Téllez Iñón
Venkatachalam Udhayakumar
Christopher Uejio
Uade Ugbomoiko
Buddy Ullman
Thomas Unnasch
Britta Urban
Joseph Urban
Julio Urbina
Jürg Utzinger
Jude Uzonna
Gustavo Valbuena
Jesus Valenzuela
Glyn Vale
Denis Valle
Olaf Valverde Mordt
Willem Van Panhuis
Frederik Van den Broeck
Alain Vanderplasschen
Luc Vanhamme
Dana Vanlandingham
Veerle Vanlerberghe
Sophie Vanwambeke
José Ronnie Vasconcelos
Pedro Vasconcelos
Nikos Vasilakis
Subhash Vasudevan
Gonzalo Vazquez-Prokopec
Raman Velayudhan
Juan Venegas
Arun Venkatesan
Jonathan Vennerstrom
Lucio Vera-Cabrera
Stefano Veraldi
Manuela Verastegui
Patricia Veras
Baptiste Vergnes
Stephane Verguet
Niels Verhulst

Liana Verinaud
Jon Vermeire
Jaco Verweij
Dario Vezzani
Alejandro Viale
Cecile Viboud
Carlos Vicente
Leda Vieira
Paulo Vieira
Marco Vignuzzi
Rafael Vilasanjuan
Sharon Villanueva
Luis Villar
Joseph Vinetz
Rodolfo Viotti
Leo Visser
Johnny Vlaminck
Carol Vlassoff
Petr Volf
Asisa Volz
Sirenda Vong
John Vontas
Carlijn Voorend
Penelope Vounatsou
Dominique Vuitton
Timothy Wade
David Wagner
Hermann Wagner
Tyler Wagner
Naomi Waithira
Peter Walden
Anthony Walker
David Walker
Stephen Walker
John Wallace
Ryan Wallace
Lance Waller
Robert Wallis
Pegine Walrad
Michael Walsh
Judd Walson
C. Wamae
Jinfeng Wang
Shi-Ping Wang
Shiwen Wang
Tian Wang
Wei-Kung Wang
Samuel Wanji
Mark Wansborough-Jones
Mark Wansbrough-Jones
Jonathan Warawa
Alon Warburg
Nicola Wardrop
Michael Ward
Robert Waterhouse
Douglas Watts
Scott Weaver
Cameron Webb
Mari Webel
Bonnie Webster
Janis Weeks
Manfred Weidmann
Gary Weil
Hana Weingartl
Philip Weinstein
Brian Weiss
Susan Welburn
Melanie Wellington
Tim Wells
Oliverio Welsh
Edward Wenger
Karl Werbovetz
Guilherme Werneck
Catherine Werts
Sheila West
T. Eoin West
A. White, Jr.
Stephen Whitehead
Lisa White
James Whitworth
Bill Wickstead
Douglas Widman
Giovanni Widmer
W. Wiersinga

Thomas Wierzba
Henry Wilde
Annie Wilkinson
Patricia Wilkins
Heather Williamson-Jordan
Brian Williams
Craig Williams
David Williams
Diana Williams
Maya Williams
Nierman William
Arve Willingham
Bridget Wills
Mary Wilson
Michael Wilson
R. Alan Wilson
Shona Wilson
William Wilson
Ken Winkel
Andrea Winkler
Gary Winslow
Elizabeth Winzeler
Dawit Wolday
Adrian Wolstenholme
Surasakdi Wongratanacheewin
Kimberly Won
Dana Woodhall
Christopher Woods
Jon Woods
Stefan Worgall
Carsten Wrenger
Elsio Wunder Jr
Jianguo Wu
XP Wu
Thomas Wynn
Lihua Xiao
Shang Xia
Zhiyong Xi
Ling Xue
Rui-de Xue
Bing Xu
Jing Xu
Wenrong Xu
PEILING YAP
Sophie Yacoub
Zaida Yadon
Pablo Yagupsky
Laith Yakob
Lydia Yamaguchi
Yoshihisa Yamano
Hiroshi Yamasaki
Tetsuya Yanagida
Kun Yang
Pengyuan Yang
S Yanow
George Yap
Vanessa Yardley
Maria Yazdanbakhsh
Jung-Yong Yeh
Yuzhen Ye
Jonathan Yoder
In-Kyu Yoon
Nobuko Yoshida
Timothy Yoshino
Neil Young
Aisha Yousafzai
Hong You
Kwok-Yung Yuen
Ruan Yuhua
Joshua Yukich
Xue-jie Yu
Arnaldo Zaha
Syed Sohail Zahoor Zaidi
Jennifer Zambriski
Keivan Zandi
Xingxing Zang
Dante Zarlenga
Jorge Zavala-Castro
Steven Zeichner
Raphael Zellweger
Anqi , Zhang
Shu Zhang
Wenbao Zhang
Yaobi Zhang

Zhijie Zhang
Bin Zhan
Yang Zhao
Elyes Zhioua
Dongsheng Zhou
Xiao-Nong Zhou
Emily Zielinski-Gutierrez
Eduard Zijlstra
Dan Zilberstein
Jakob Zinsstag
Kate Zinszer
Richard Zuerner
Ines Zulantay
Ellen Zwarthoff
henri agut
Jose Maria alunda
chen chen
kenneth coggeshall
Joao da Rosa
Alexandre da Silva
Edson da Silva
Ruklanthi de Alwis
Zoilo de Camargo
William de Glanville
Sybren de Hoog
Maria de Lourdes Munoz
Isabel de Miranda Santos
Camila de Oliveira
Rafael de Roodt
Aravinda de Silva
Nilanthi de Silva
Wanderley de Souza
Juana del Valle Mendoza
Margriet den Boer
maha eissa
daozhou gao
richard guerrant
susan hills
Andrea keane-myers
klitos konstantinidis
thomas marrie
Luz moyano
pierre nouvellet
juan olano
antonio osuna
lynn schriml
nicholas sibinga
gustave simo
Jan ter Meulen
Wim van Brakel
Johan van Griensven
Martijn van Hemert
Lisette van Lieshout
Ilana van Wyk
Wendy van de Sande
Petra van den Doel
Andrew van den Hurk
Gert van der Auwera
Ashutosh verma
Georg von Samson-Himmelstjerna
Lorenz von Seidlein
Esther von Stebut
Catherine walton
Peigang wang
james whitehorn
Marion woods
edward young
xichen zhang
David Šmajs
